# Classifying hand sensorimotor functions of the chronic kidney disease patients using novel manual tactile test and pinch-holding-up activity

**DOI:** 10.1371/journal.pone.0219762

**Published:** 2019-07-11

**Authors:** I-Te Tu, Yu-Shiuan Cheng, Pu-Chun Mo, Hsiu-Yun Hsu, Li-Chieh Kuo, I-Ming Jou, Fong-Chin Su

**Affiliations:** 1 Department of Biomedical Engineering, National Cheng Kung University, Tainan, Taiwan; 2 Division of Nephrology, Department of Internal Medicine, Chi Mei Medical Center, Liouying, Tainan, Taiwan; 3 Department of Occupational Therapy, National Cheng Kung University, Tainan, Taiwan; 4 Department of Orthopedics, E-Da Hospital, Kaohsiung, Taiwan; 5 Medical Device Innovation Center, National Cheng Kung University, Tainan, Taiwan; University of Ottawa, CANADA

## Abstract

Hand function deterioration brings about inconvenience to the daily lives of the chronic kidney disease patients. However, a full spectrum of hand function examination is absent. Therefore, this study aimed to classify the hand sensorimotor functions of the chronic kidney disease patients using the novel sensorimotor assessment tools, manual tactile test (MTT) and pinch-holding-up activity (PHUA) test, and explore the feasibility in comparison with traditional evaluations in the clinical practice. 68 stage-5 chronic kidney disease patients and 50 healthy subjects were recruited in this study. A series of conventional evaluations and two novel hand function tools, manual tactile test and pinch-holding-up activity test were conducted from the perspective of hand dexterity, sensory input threshold, force generation and sensorimotor control. Independent t-test was used to find out group differences and the receiver operating characteristic curve was used to determine accuracy of the tests. In our results, significant reduction of hand dexterity, sensory input, force generation and sensorimotor control was found in patients from an overall perspective. This trend was discovered to be the same when dividing the subjects into the old and young age group. From the receiver operator characteristic curves, nearly all the areas under the curve of all tests were over 0.8. The novel evaluation tools, the manual tactile test and pinch-holding-up activity, were found to have comparable or even better accuracy than the traditional ones. The shape and weight subtests of the manual tactile test displayed the highest accuracy. To sum up, by incorporating the novel and conventional assessment tests, this study built up the fundamental understanding of the hand functions in multiple dimensions and consolidate the clinical merits of applying the two novel tools, manual tactile test and pinch-holding-up activity, on chronic kidney disease patients.

## Introduction

Chronic kidney disease (CKD) has an estimated prevalence of around 8.2% worldwide and the actual number varies with different areas.[1] It has been a major problem for many people especially for those in stage five. On top of that, among various complications, peripheral neural dysfunction has brought a huge impact on the daily lives of patients with CKD. Mononeuropathies are commonly seen in patients with CKD, especially forearm nerves. Moreover, patients undergoing chronic dialysis are predisposed to mononeuropathies.[2] Ulnar nerve syndrome and carpal tunnel syndrome are all possible complications with a high incident rate, leading to finger numbness, hand muscle atrophy or adhesion of tendon sheaths.[3–7] Though mononeuropathies are quite common clinically, there is limited knowledge from previous studies, especially in comprehensive hand function assessment.

Plenty of hand function assessment tools have been developed in many forms including questionnaires, certain motion, and equipment. Some have been utilized in CKD patients. Daily activity related tests were performed including the Sollerman test, Grip Function test, Hand Functional Index, and Duruöz Hand Index.[8–10] In addition, decrease in handgrip and pinch strength are shown in patients measured by a hand dynamometer and pinch gauge.[8] Moreover, to evaluate fingertip dexterity, the Purdue pegboard test was used on patients and it was negatively correlated with the Duruöz Hand Index.[8] Although hand function assessment tools have been regularly used, most of the modalities mentioned above are still remained to be subjective and qualitative.

Recently, Manual tactile test (MTT) was developed to evaluate the synthesizing sensibility by manually exploring different kinds of objects by hands. MTT has shown high testing reliability, validity and accuracy for patients of carpal tunnel syndrome (CTS)[11, 12] and peripheral nerve injury.[13, 14] The MTT has shown to capture functional improvement in the course of nerve regeneration and mild to medium correlation with other traditional hand function tests was found.[13] In addition, an apparatus composed of load cells and an accelerometer was built for pinch-holding-up activity (PHUA) test. The adjusting ability of pinch force along with temporal parameters are used to assess sensorimotor control of the hand, displaying the ability to perform smooth, timely and skillful movements. It was used on patients with diabetes mellitus (DM)[15, 16] and peripheral nerve injury.[14] From the increase of the pinch and force ratio as well as percentage of the maximum pinch force, sensorimotor control was found to significantly decline in these patients. Higher force ratio and higher percentage of the maximum pinch force indicated more pinch force was employed with respect to the load as well as more proportion of the maximum force was needed to be generated by the subject to control the lift movement. Since the seemingly simple lift movement was actually a consequence of dynamic neuromuscular control[17, 18], increase in these two parameters usually represented more effort was required to complete the task and an interruption of the sensorimotor control.[19, 20] Furthermore, the PHUA was also combined with visual feedback to successfully assess and re-educate force modulation for stroke patients with sensory deficits.[21] Both the MTT and PHUA test have been tested to be reliable tools to evaluate hand function with better accuracy and integrated information than the traditional tools. However, no study has implemented them on CKD patients and compared with the healthy. More diverse and objective information on active sensory input and sensorimotor control can be retrieved from the two tests and perhaps they are able to better characterize the hand function deterioration in CKD patients.

Since the disturbance of hand functions negatively impacts the quality of life of the CKD patients, it is crucial to have an early identification and appropriate management. Although some conventional tools have been used to assess the hand function reduction, the active sensation and sensorimotor control have not been investigated for the CKD patients. Therefore, this study intended to utilized the MTT and PHUA in comparison with conventional tools to look into multiple aspects of hand function changes in CKD patients. Furthermore, the sensitivity and specificity of the MTT and PHUA were also examined to diagnostic feasibility in the clinical practice. It was hypothesized that CKD patients would show significantly lower hand functions than the healthy and the MTT and PHUA test would have a higher or equal accuracy in identifying the hand function deterioration than the traditional tests.

## Materials and methods

### Participants

A total of 68 stage-5 (glomerular filtration rate lower than 15 mL/min) CKD patients were recruited as the experiment group and 50 healthy adults were included as the control group. The following conditions were used as exclusion criteria: (1) traumatic nerve injuries of the upper limbs, (2) trauma to the hand or congenital anomalies of the wrist and hand, (3) skin infections or disease, (4) known vascular complications of DM, such as stroke may have compromised the physical integrity of the patient, (5) grade 2 or higher arterial hypertension (>160/100 mmHg) or (6) cognitive deficits. Among the patients, five patients had a poor eyesight and were unable to see clearly of the elements of the Purdue Pegboard test, therefore, these five patients did not undergo the Purdue Pegboard test. Moreover, one patient did not complete the grip force and PHUA due to an early leave of an urgent personal affair. In addition, after being fully notified of the purpose of the study, detailed experimental procedures, risks and benefits, compensations, and contact information, all subjects signed a consent form approved by the Institutional Review Board of the Chi Mei Medical Center. The Institutional Review Board of the Chi Mei Medical Center specifically approved this study (10610-L02).

### Experimental design

The demographic information and regular clinical data were recorded first. After that, the subjects went through a set of tests for hand function in a random sequence, including sensibility, strength, sensorimotor control. Details of each test are shown below.

#### Semmes-Weinstein Monofilament (SWM) test

The SWM test was used to evaluate the sensory neuropathy and determine the touch-pressure threshold of the hands in this study. When applying the test, the examiner placed the monofilament perpendicular to the skin surface and pressed downward to bend the monofilament to exert a constant force (due to the elastic property of the nylon filament) onto the skin area of the fingertip. The monofilaments are labeled with a numerical score (higher score represents a thicker monofilament), which is a log to the base ten of the force in tenths of milligrams. Different thickness of the monofilament was used one by one to find the minimum touch-pressure received by the subject. The hand was static on the table and the thumb and little finger were tested for different nerve innervation regions.

#### Purdue pegboard test

The Purdue pegboard test was used to test the finger dexterity of the subjects. Participants were instructed to arrange as many pins and assemble pins, washers, and collars as possible within a fixed duration. The assessment consisted of unilateral pin insertion (dominant and non-dominant hand), bilateral pin insertion and assembly subtest. The duration of the assembly subtest was 60 seconds and the other three subtests were 30 seconds, respectively.

#### Manual tactile test (MTT)

**Fig 1:**
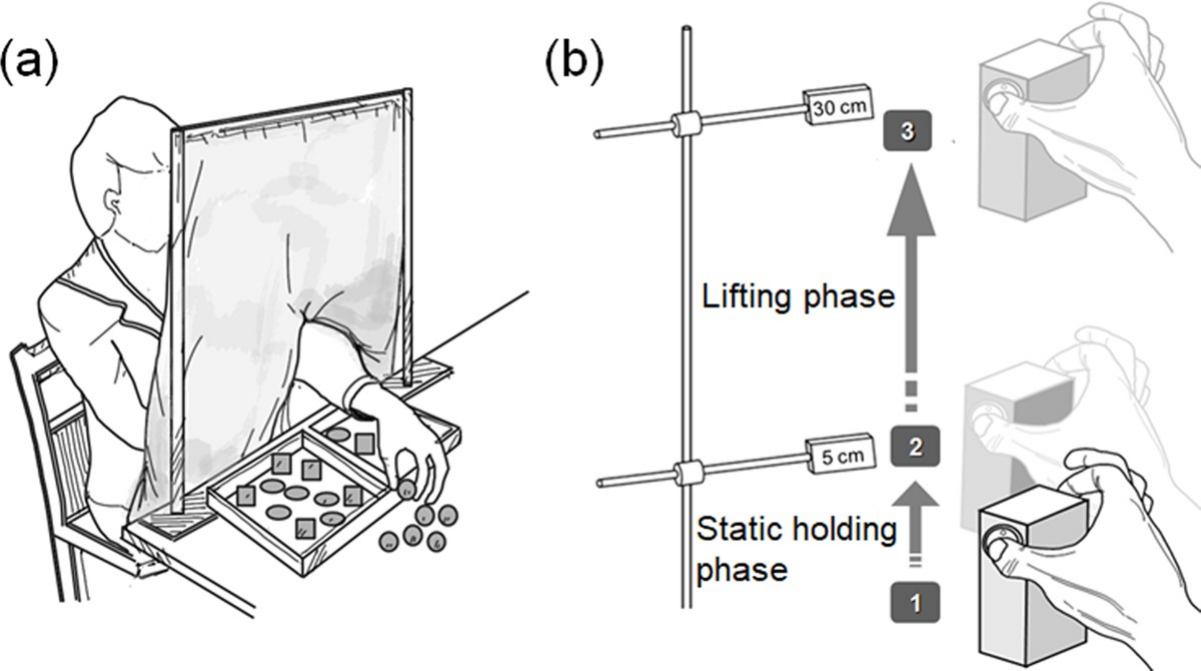
Illustration of the novel evaluation tools. (A) manual tactile test (B) pinch-holding-up activity test.

The MTT test was used to assess the sensory function of the subjects, including the Barognosis, Roughness and Stereognosis test. (1) Barognosis test: Objects which are made of plastic material with identical shape (cylindrical) and size, 5.4 cm in diameter and 10 cm in height. Three different cylinders with same shape and size are made of 3 different weights, 150, 225, and 300 grams, for the subject to differentiate. (2) Roughness test: Plastic cubes (2.5 cm in width) coated with three different levels of roughness are used to detect the roughness perception. The degrees of roughness are divided into 3 types of cubes including a smooth surface, a mild rough surface (plastic surface carved with a 1 mm × 1 mm grid), and the roughest surface (plastic surface carved with 2 mm × 2 mm grid). For each roughness, 6 cubes are used, resulting in a total of 18 cubes. (3) Stereognosis test: Three objects with different shapes, namely cubes, ellipsoids, and spheroids, made of plastic are used to evaluate form perception. All the objects are identical with regard to their weight and the roughness of their surfaces. There are 6 objects for each shape, and a total of 18 objects are used for this test. In each of the 3 tests, the time to complete sorting different objects blindly was recorded (Fig 1A). The detailed testing procedures of the three subtests of MTT have been described in our previous work.[13, 14]

#### Pinch-holding-up activity (PHUA) test

The PHUA test was utilized to examine sensorimotor control through a simple lifting movement with a pinch apparatus (Fig 1B). The apparatus is composed of the following parts: (1) a specially designed steel cuboid (6.0*4.5*9 cm); (2) two 6-axis load cells (Nano-25, ATI Industrial Automation, Apex, NC) that are mounted in the cuboid to detect the pinch and lifting forces exerted by the thumb and index finger; and (3) a tri-axial analog accelerometer (Model 2412, Silicon Designs, Inc., Issaquah, WA) that is used to record the acceleration of the pinch device in space during the test. Subjects were instructed to hold the apparatus and execute the following steps: (1) use the pulps of the thumb and index finger to pinch and lift the apparatus to around 5 cm above the table for 5 seconds; (2) then lift the apparatus to a height of 30 cm at a self-paced speed and stop for around 3 seconds; (3) slowly lower to the starting position. The formal procedure was repeated for at least 5 times after 3 practice trials to make sure the subjects performed the most natural movement. The pinch and load force were recorded during the whole process for further analysis. Finally, for the normalization purpose, a maximal pinch force (Static FP_Peak_) was tested with the subject performing a pinch at a height of 30 cm above the table. Static FP_Peak_ also showed the maximum capability of the subject to generate pinch force.

### Data analysis

**Fig 2:**
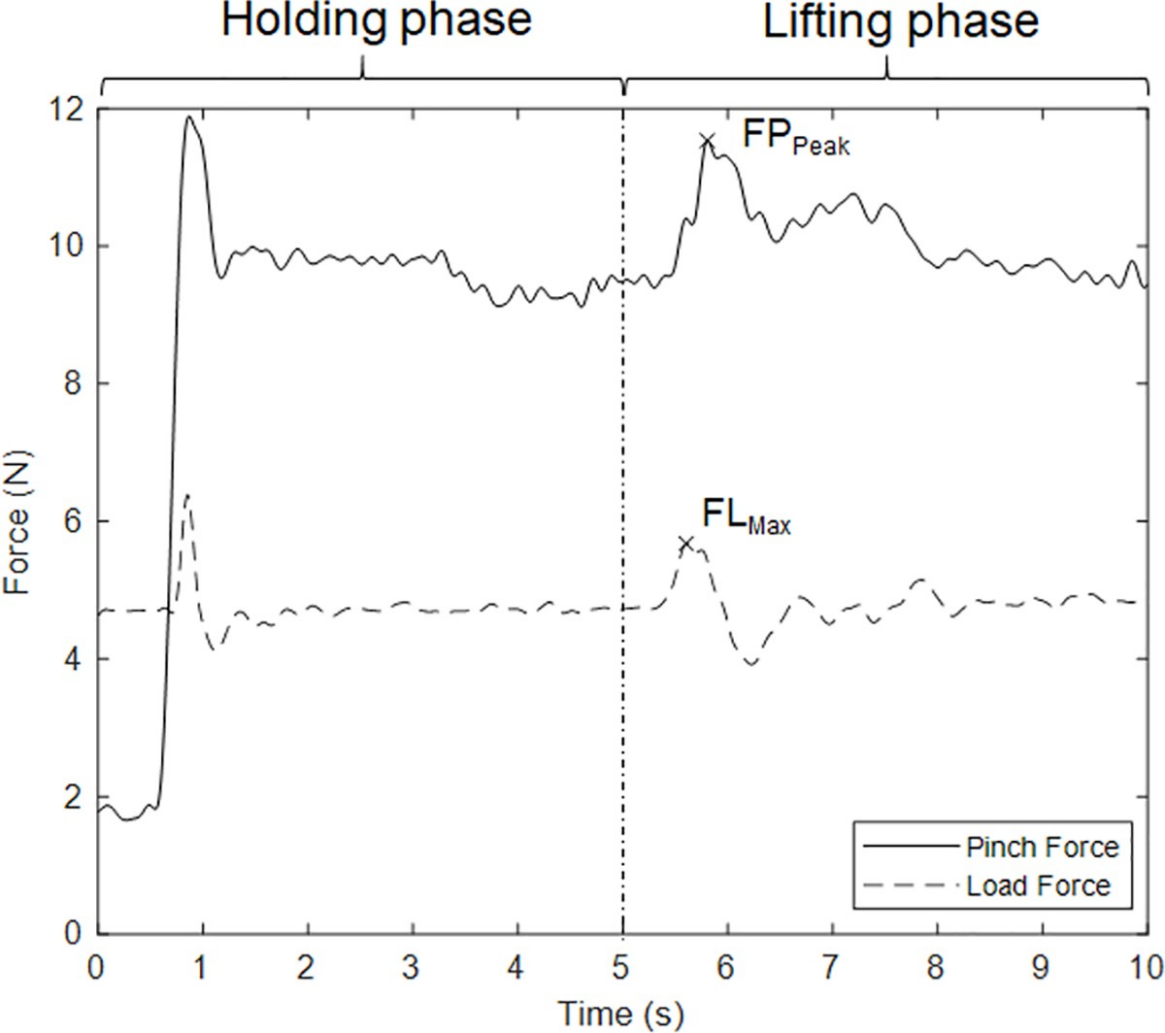
Demonstration of the pinch and load force VS time graph. The solid line represents the pinch force and the dashed line represents the load force.

From the PHUA test, the resultant load and pinch force were calculated through the root mean square from the three separate axes. Five parameters were then calculated using the resultant forces (sum of the forces from the thumb and index finger) and time curves (Fig 2). (1) FP_Peak_: maximum pinch force during the lifting phase in the PHUA test. (2) FL_Max_: maximum load force during the lifting phase. (3) FR: the force ratio between FP_Peak_ and FL_Max_. (4) % Static FP_Peak_: maximum pinch force normalized by the static maximal pinch force with a unit of percentage. (5) FR_Norm_: the ratio between % Static FP_Peak_ and normalized FL_Max_ (normalized as multiples of acceleration of gravity) to combine force ratio with normalization.

### Statistical analysis

Prism 5.0 GraphPad was used for statistical analysis. The descriptive statistics were calculated for each of the parameters from the evaluation tools. To find out the alteration of multiple aspects of hand functions in CKD patients, independent t-test was utilized to compare the differences between the patient and the control group. In addition to the overall comparison, the subjects were also divided into 2 groups based on age (above or below 55 years old). Independent t-test was also performed to the two age groups respectively to see the differences between the patient and the control to make a more detailed comparison regarding age. Moreover, the receiver operating characteristic (ROC) curve was built from the sensitivity and specificity values of the tests. The area under the ROC curve was used to determine the accuracy of the tests. The optimized cutoff values to differentiate the patients from the healthy in each test were determined by the Youden’s index defined by (sensitivity + specificity—1). A *p*-value of 0.05 was set as the significance level.

## Results

**Fig 3:**
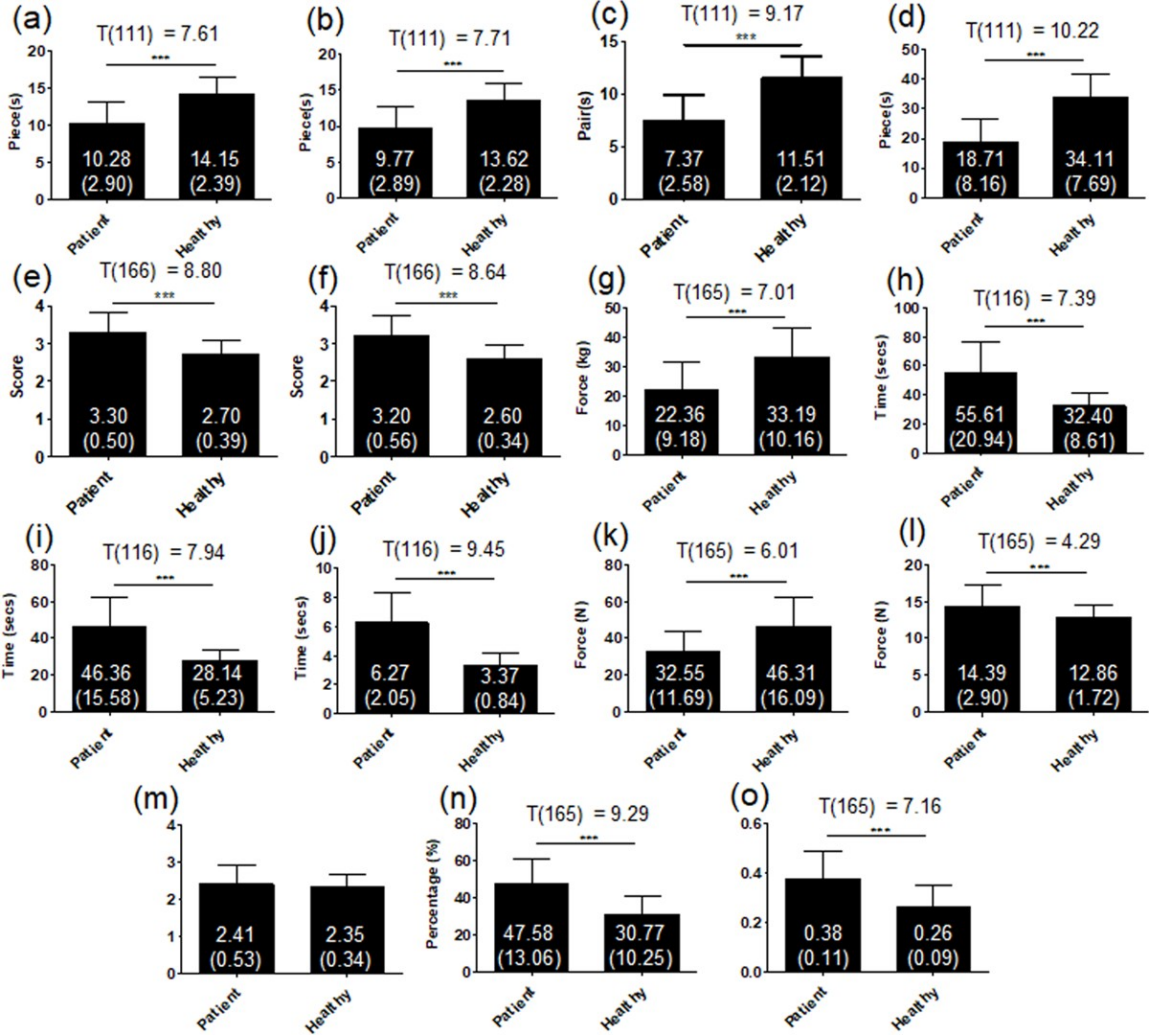
Overall comparison different hand function tests between the patients and the healthy. ***p < .001. Bars are plotted with mean and standard deviation. Each subgraph represents the following: Purdue Pegboard test: (A) dominant hand, (B) non-dominant hand, (C) both hands and (D) assembly; SWM test: (E) thumb and (F) little finger; (G) Grip force; MTT: (H) roughness, (I) shape and (J) weight subtest; PHUA parameters: (K) Static FP_Peak_, (L) FP_Peak_, (M) FR, (N) % Static FP_Peak_ and (O) FR_Norm_.

The basic information, including age, gender, and dialysis duration, of the subjects is shown in Table 1. Overall, the patients displayed decreased ability in each of the hand function assessment tool. In the Purdue Pegboard test, patients completed significantly fewer pieces and pairs than the healthy in all four subtests (*p* < .001, Fig 3A–3D). In the SWM test, a significantly higher score of both fingers was shown in the patient group (*p* < .001, Fig 3E and 3F), representing lower finger perception on touch-pressure. In the grip force measurement, the maximal grip force of the patients was also significantly lower than the healthy (*p* < .001, Fig 3G). Furthermore, in the MTT, the patients spent significantly more time to complete the task than the healthy in all three subtests (*p* < .001, Fig 3H–3J). In the PHUA test, significantly lower Static FP_Peak_ was found in the patient group (*p* < .001, Fig 3K). Moreover, the patients also showed larger FP_Peak_, % Static FP_Peak_ and FR_Norm_ than the healthy (*p* < .001, Fig 3L, Fig 3N and 3O). However, no difference was found in FR (Fig 3M).

**Table 1:** Basic information of the experiment and control group subjects.

	Patients	Healthy
Sample size	68	50
Age	60.19±9.38 (Range: 29–88)	53.14±16.01 (Range: 21–81)
Males	43	30
Females	25	20
> = 55 years old	50	30
< 55 years old	18	20
Dialysis Duration (years)	5.44±3.82	

Data are Mean±SD

**Fig 4:**
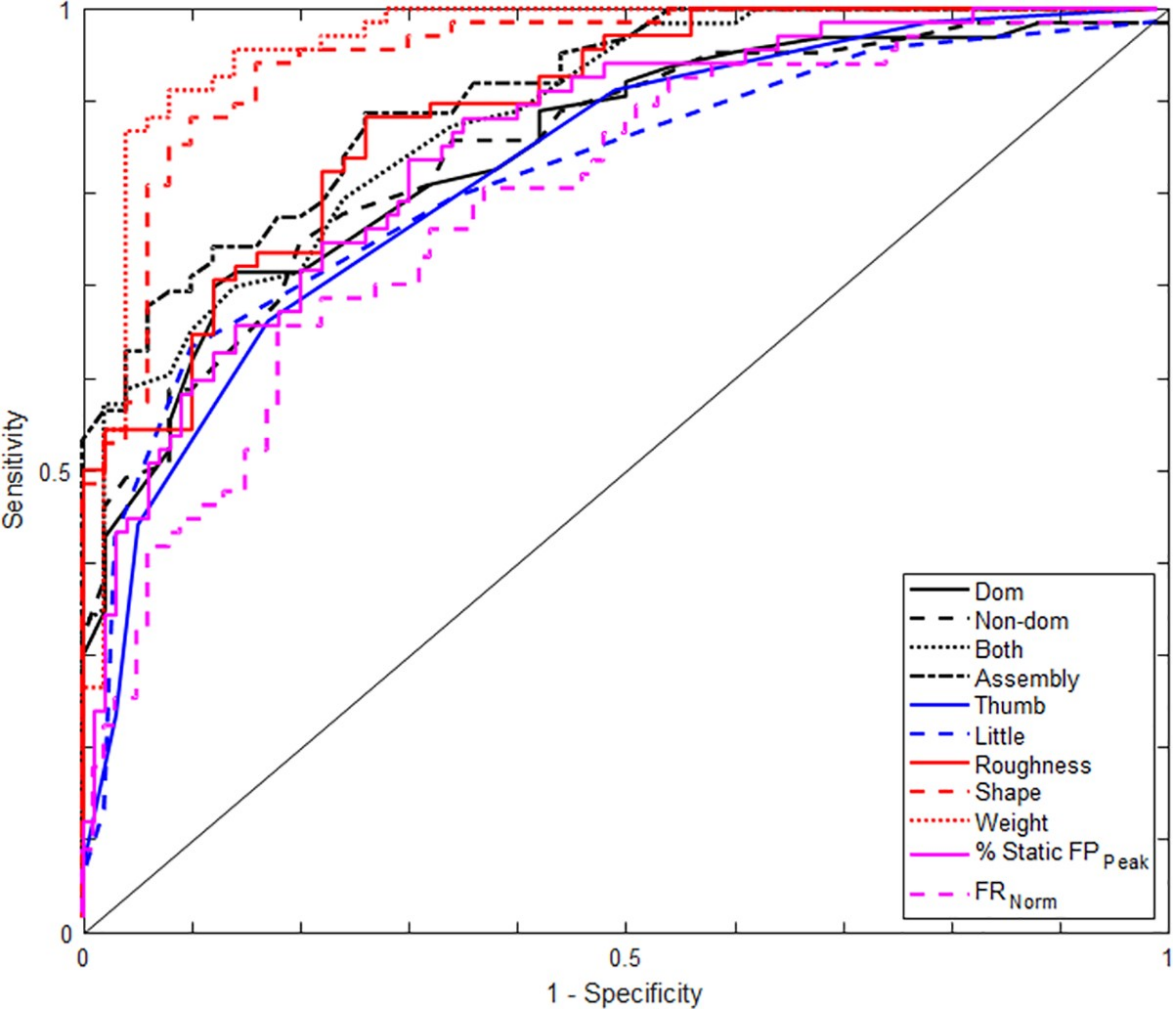
ROC curves of each hand function assessment tools. Black lines are from the Purdue Pegboard test, blue lines are from the SWM test, red lines are from the MTT and purple lines are from the PHUA test.

In the age-divided comparison results, the trend of each parameter was basically the same as the overall comparison. The patients generally displayed a worse performance in all the hand function evaluations than the healthy including lower scores in Purdue, larger pressure thresholds in SWM, lower static grip and pinch force, more time to complete the active sensory differentiation tasks in the MTT, and worse force modulation in the PHUA, in both of the age group. The detailed statistical outcome is shown in Table 2 and Table 3. In addition, the ROC curves of each evaluation tool were generated using the overall data and plotted in Fig 4. The area under the curve (AUC) was calculated as well as the optimized cutoff value, sensitivity, and specificity. They are shown in Table 4. All the tested items were found to have an AUC larger than 0.8 except for the FR_Norm_ was found to be 0.79. Some subtests were discovered to have an AUC over 0.9 including the assembly subtest of Purdue Pegboard test, shape and weight subtests of the MTT. Furthermore, the MTT was found to have better accuracy in predicting hand function loss of hemodialysis patients than the conventional tools. On the other hand, the PHUA demonstrated comparable accuracy with the conventional tools.

**Table 2:** Statistical outcomes of each test of subjects above or equal to 55 years old.

> = 55 years old		Patients	Healthy	*p*-value	t-value & df
Purdue	Dominant Hand	9.55±2.66	12.99±2.10	< .001***	T(73) = 5.95
	Non-Dominant Hand	9.10±2.66	12.48±2.10	< .001***	T(73) = 5.85
	Both Hands	6.79±2.29	10.43±1.89	< .001***	T(73) = 7.24
	Assembly	16.43±7.13	30.69±7.16	< .001***	T(73) = 8.47
SWM	Thumb	3.35±0.49	2.69±0.42	< .001***	T(108) = 7.61
	Little	3.26±0.54	2.59±0.36	< .001***	T(108) = 7.71
Grip Force (kg)		20.31±8.76	31.20±8.61	< .001***	T(108) = 6.55
MTT	Roughness (secs)	59.31±20.98	35.84±8.57	< .001***	T(78) = 5.83
	Shape (secs)	48.77±16.41	29.49±5.31	< .001***	T(78) = 6.23
	Weight (secs)	6.72±2.15	3.64±0.88	< .001***	T(78) = 7.46
PHUA	Static FP_Peak_ (N)	31.12±9.51	45.24±17.07	< .001***	T(108) = 5.21
	FP_Peak_ (N)	14.28±3.01	13.20±1.81	.022*	T(108) = 2.32
	FR	2.38±0.56	2.44±0.35	.552	T(108) = 0.60
	% Static FP_Peak_ (%)	48.86±13.54	32.66±11.05	< .001***	T(108) = 6.91
	FR_Norm_	0.39±0.12	0.28±0.09	< .001***	T(108) = 5.01

Data are Mean±SD; df = degree of freedom

***p < .001

*p < .05

**Table 3:** Statistical outcomes of each test of subjects below 55 years old.

< 55 years old		Patients	Healthy	*p*-value	t-value & df
Purdue	Dominant Hand	12.11±2.71	15.88±1.63	< .001***	T(36) = 5.26
	Non-Dominant Hand	11.44±2.83	15.33±1.23	< .001***	T(36) = 5.59
	Both Hands	8.83±2.75	13.13±1.21	< .001***	T(36) = 6.36
	Assembly	24.43±7.93	39.23±5.30	< .001***	T(36) = 6.84
SWM	Thumb	3.16±0.49	2.72±0.33	< .001***	T(56) = 4.03
	Little	3.02±0.58	2.61±0.30	< .001***	T(56) = 3.55
Grip Force (kg)		28.39±7.84	36.17±11.59	.015*	T(55) = 2.53
MTT	Roughness (secs)	45.34±17.53	27.24±5.70	< .001***	T(36) = 4.38
	Shape (secs)	39.67±10.75	26.13±4.51	< .001***	T(36) = 5.16
	Weight (secs)	5.04±1.04	2.96±0.56	< .001***	T(36) = 7.80
PHUA	Static FP_Peak_ (N)	36.77±16.17	47.92±14.57	.013*	T(55) = 2.56
	FP_Peak_ (N)	14.72±2.58	12.35±1.45	< .001***	T(55) = 4.44
	FR	2.48±0.43	2.22±0.27	.008**	T(55) = 2.77
	% Static FP_Peak_ (%)	43.79±11.02	27.93±8.26	< .001***	T(55) = 5.98
	FR_Norm_	0.35±0.10	0.24±0.07	< .001***	T(55) = 5.08

Data are Mean±SD; df = degree of freedom

***p < .001

**p < .01

*p < .05

**Table 4:** Outcomes from the ROC curves and Youden’s index.

		AUC	Optimized Cutoff Value	Sensitivity (%)	Specificity (%)
Purdue	Dominant Hand	0.846	11.5	70	88
	Non-Dominant Hand	0.846	11.5	75	80
	Both Hands	0.881	9.167	70	86
	Assembly	0.906	29.67	89	74
SWM	Thumb	0.823	3.025	66	83
	Little	0.817	3.025	63	90
MTT	Roughness	0.889	36.44	88	74
	Shape	0.947	33.31	88	90
	Weight	0.961	4.298	91	92
PHUA	% Static FP_Peak_	0.848	35.96	84	70
	FR_Norm_	0.790	0.337	66	82

## Discussion

This study has successfully utilized both the conventional and novel evaluation tools and found decreased hand functions in multiple aspects in the patients, which was in accordance with the hypothesis. To begin with, from the Purdue Pegboard test results, the patients had a significantly lower number of completed pieces and pairs than the healthy in all four subtests. In the Purdue Pegboard test, different types of hand dexterity, from simple single-handed to complicated dual-handed tasks, were examined. On top of that, hand dexterity was not only influenced by the motor function but also the sensory capability as well. Therefore, the subjects’ ability to complete tasks requiring delicate finger manipulation has been impaired due to the effect of hemodialysis on nerve functions. The Purdue results of the patients were in accordance with a previous study in which the pin number was found to be 12.00±2.18, 11.00±2.18 and 20.99±5.31 in the dominant, non-dominant hand and assembly task, respectively.[8]

Moreover, the SWM results demonstrated that the score of both thumb and little finger of the patients was significantly higher than the healthy, showing decreased sensory input ability of the touch-pressure. By applying a different thickness of filaments to the cutaneous surface of the finger, self-perception of the filament was used to identify the threshold of the sensory input of the touch-pressure. The SWM score of the CKD patients (3.30±0.50) were similar with the CTS patients (3.45±0.38) but the sensory perception was better than the peripheral nerve patients (4.27±0.48).[11–14] Although the passive stimulus from the SWM was shown to be a highly subjective,[22] it was still undeniable that the patient's finger sensibility was compromised due to the CKD. Furthermore, the maximal grip force results also demonstrated that the patients were not able to exert as much force as the healthy by their hands. Force generation of a grip is originated from the muscles around the hand as well as flexor muscles in the forearm. Therefore, it was shown that hemodialysis might directly or indirectly jeopardize the muscle function as the driving source of the hand force generation. Previously, muscle atrophy has been found in patients receiving hemodialysis with smaller contractile muscle cross-sectional area as well as lower physical strength and activities.[23, 24] The overall mean grip force of the CKD patients in this study (22.03±9.51 kg) was found to be similar with that in a previous study (25.0±11.4 and 22.8±12.1 kg).[9]

On the other hand, outcomes from the two novel evaluation tools also indicated a drop in the sensorimotor control. First, from the MTT, more time was required for the patients to complete the tasks in all the subtests, representing during the sorting process, the patients spent more time and effort in processing the sensory input of roughness, shape and weight through the digits. Texture and shape perception decline has been reported in the median or ulnar nerve injury patients.[25] However, the overall performance of the texture and shape subtests (55.61±20.94 and 46.36±15.58 seconds) of the CKD patients were discovered to be better than patients of the peripheral nerve injury (69.6±37.6 and 53.7±38.0 seconds) as well as stroke (73.8±29.7 and 49.0±19.5 seconds). In addition, the sensing ability of weight, which demands integration of multiple forms of information, also served as a crucial factor in comprehensive sensory assessment. Patients with sensory deficits were also found to have affected weight discrimination.[26] It was shown that the CKD patients displayed longer time to discriminate weight differences (6.27±2.05 seconds) than other neurological diseases such as the CTS (3.11±1.02 seconds) and stroke (5.1±1.1 seconds),[11, 12, 27] indicating that the CKD might pose a more serious threat to the weight sensory input.

Moreover, the overall performance of sensorimotor control of the patients from the PHUA test was lower than that of the healthy. To go into a deeper analysis of the functional status, each parameter reflected a different feature of the motion. To begin with, smaller Static FP_Peak_ of the patients indicated weaker muscle force generation of the fingers. In addition, larger FP_Peak_ and % Static FP_Peak_ of the patients represented that they were using more force and effort to control the lifting movement. This could result from the instability and a sense of insecurity caused by the poorer sensorimotor function. It was found that people with less sensory modulation tended to exert excessive force or have clumsy manipulation during a task-based action.[17–20] % Static FP_Peak_ of the CKD patients (47.58±13.06%) was shown to be higher than patients of peripheral nerve injury (42.6±15.7%) and DM (35.52±10.17% and 33.57±9.6%),[14, 15] demonstrating larger negative impact on the pinch force modulation.

Furthermore, the adjustment of pinch force in relation to the loading during the lifting phase also played an essential role since such a change was an automated response to achieve stability.[17, 28] Therefore, larger FR and FR_Norm_, which represented the relationship between pinch and load force, of the patients were discovered to be larger than the healthy, indicating lower efficiency of dynamic force modulation. In addition to the original parameter FR described in the previous studies,[15, 21] the information of the normalized magnitude of pinch force control (% Static FP_Peak_) was combined with the FR, producing a new parameter of FR_Norm_ with more abundant manifestation. In the overall comparison, FR_Norm_ was shown to be more effective in capturing the sensorimotor control capability in terms of CKD with a significant difference than the FR, which did not show any significant difference. Increase in FR_Norm_ of the CKD patients was in accordance with the previous studies, which showed larger FR in patients with CTS, peripheral nerve injury, stroke and DM as well.[12, 14, 15, 27]

Age has been widely-known to affect hand functions and tactile pressure threshold according to much of the previous evidence.[11, 29–32] Reduced abilities to detect object weight, 3D shape and texture have been discovered in older people.[33–35] Therefore, two groups with different ages were separated to eliminate the potential extra interference of the outcome. From the results, the trend of all the parameters of each tool was the same as the overall comparison, suggesting that the hand dexterity, sensory input, muscle force generation and sensorimotor control of the CKD patients were all indeed deteriorated regardless of which age group.

Last but not least, according to the ROC curves with different colors indicating different evaluation tools, two of the MTT subtests (red lines) seemed to have a better total performance with curves further away from the reference line, while the PHUA test showed a similar trajectory with the conventional tools. To look into deeper, the MTT showed the highest overall AUC and better sensitivity and specificity. In general, with nearly all the AUC above 0.80, the optimized cutoff values can be used to help diagnose different aspects of the hand functions reduction in hemodialysis patients. Although both the SWM and MTT might seem like sharing the same goal of examining the sensory function, the MTT was aimed to test the active touch senses while the SWM was used to test the passive ones. Aside from the fact that most of the daily activities need active perception from self-generated movements, the diminished transmission of tactile input and insufficient acquirement of the sensory data[36, 37] under the passive touch are also reasons that the MTT has its unique advantage over the SWM. The MTT involves a dynamic pick and sorting process that is closer to the scenarios in the daily activities, therefore, it is perhaps more accurate in reflecting the perception capabilities of the subjects.

Several limitations existed in this study. To begin with, the subjects were recruited in only one geological location, therefore the population was more homogenous. In addition, some of the tests in this study required active hand movement, therefore severer patients with insufficient ability to execute the tasks were unable to be included. Moreover, the actual peripheral nerve status was not included in this study. This should be incorporated in the future so as to build the connection between the nerve condition and hand function.

## Conclusions

Multiple dimensions of hand functions have been successfully evaluated using both traditional and novel evaluation tools, MTT and PHUA. Overall, a significant decrease in dexterity, sensory input, and sensorimotor control have been discovered in CKD patients in comparison with the healthy. After dividing the subjects into two groups according to the age, the trend of the hand function reduction still remained unchanged. Moreover, based on the ROC curves, the novel evaluation tools displayed an equal or even better overall accuracy than the conventional ones. Two subtests of the MTT was found to demonstrate the highest AUC. This study could serve as baseline data with comprehensive hand function assessments for clinicians to help identify the deterioration of hand functions of the CKD patients.
